# Double negative T cells mediate Lag3-dependent antigen-specific protection in allergic asthma

**DOI:** 10.1038/s41467-019-12243-0

**Published:** 2019-09-18

**Authors:** Dan Tian, Lu Yang, Song Wang, Yanbing Zhu, Wen Shi, Chunpan Zhang, Hua Jin, Yue Tian, Hufeng Xu, Guangyong Sun, Kai Liu, Zhongtao Zhang, Dong Zhang

**Affiliations:** 1 General Surgery Department, Beijing Friendship Hospital, Capital Medical University, Beijing, 100050 China; 2 Experimental and Translational Research Center, Beijing Friendship Hospital, Capital Medical University, Beijing, 100050 China; 3 Beijing Clinical Research Institute, Beijing, 100050 China; 4 Beijing Key Laboratory of Tolerance Induction and Organ Protection in Transplantation, Beijing, 100050 China; 5 National Clinical Research Center for Digestive Diseases, Beijing, 100050 China

**Keywords:** Antigen presentation, Immunosuppression, T cells, Asthma

## Abstract

Allergic asthma is an inflammatory disorder of the airway without satisfactory traditional therapies capable of controlling the underlying pathology. New approaches that can overcome the detrimental effects of immune dysregulation are thus desirable. Here we adoptively transfer ovalbumin (OVA) peptide-primed CD4^−^CD8^−^ double negative T (DNT) cells intravenously into a mouse model of OVA-induced allergic asthma to find that OVA-induced airway hyperresponsiveness, lung inflammation, mucus production and OVA-specific IgG/IgE production are significantly suppressed. The immunosuppressive function of the OVA-specific DNT cells is dependent on the inhibition of CD11b^+^ dendritic cell function, T follicular helper cell proliferation, and IL-21 production. Mechanistically, Lag3 contributes to MHC-II antigen recognition and trogocytosis, thereby modulating the antigen-specific immune regulation by DNT cells. The effectiveness of ex vivo-generated allergen-specific DNT cells in alleviating airway inflammation thus supports the potential utilization of DNT cell-based therapy for the treatment of allergic asthma.

## Abstract (web summary)

Allergic asthma symptoms may be controlled, but currently no effective therapy exist to address the underlying pathology. Here the authors show, using mouse model of adoptive cell transfer, that CD4^-^CD8^-^ T cells can suppress the function of dendritic cells and T follicular helper cells via Lag3 to provide allergen-specific protection from asthma.

## Introduction

The prevalence of allergic diseases and asthma is increasing worldwide, which is a major global challenge that threatens human health and economies. Moreover, the complexity and severity of allergic diseases, including asthma, continue to increase especially in children and young adults, who are bearing the greatest burden of these trends^1,2^. Allergic asthma is characterized by hyperresponsiveness, mucus production, inflammatory cell accumulation, and allergen-specific IgE secretion. The current most commonly used effective regimen is corticosteroid treatment, but patients often report unwanted long-term side effects^3^. The development of new approaches with better efficacy and fewer side effects is imperative to improving clinical treatment.

Th2 cells are considered to be the initial CD4^+^ T cells secreting pro-Th2 inflammatory cytokines in asthma^4,5^. This pathological response is prompted by allergen-presenting dendritic cells (DCs)^6,7^. Moreover, a compelling number of reports have indicated that T follicular helper (Tfh) cells play an unexpected role in the pathogenesis of allergic asthma^8,9^. T cell subsets with regulatory functions have been discovered and applied to the treatment of autoimmune and allergic diseases^10^.

Double negative T cells (DNT cells) are unique antigen-specific regulatory T cells that express granzyme B and perforin but not Foxp3 and that were discovered in recent decades^11,12^. Although DNT cells only account for 1–3% of total T lymphocytes in the peripheral blood and lymphoid organs of humans and mice, these cells are essential for maintaining immune system homeostasis^13,14^. Our previous studies demonstrated that CD4^+^ T cells are converted to DNT cells, which can significantly suppress CD4^+^CD25^−^ T cells, mainly through the perforin/granzyme B pathway^15^. The adoptive transfer of DNT cells can prevent and reverse the onset of autoimmune diabetes and prolong islet graft survival while preserving antigen specificity^16,17^. However, DNT cells lack the CD4 molecule, which is a critical coreceptor of the TCR that assists the TCR in interacting with MHC-II; the mechanism that allows DNT cells to recognize MHC-II and maintain antigen specificity is still unknown.

Here, we demonstrate that the adoptive transfer of DNT cells ameliorates ovalbumin (OVA)-induced airway hyperresponsiveness, lung inflammation, mucus production, inflammatory cell accumulation, and OVA-specific IgG/IgE production while preserving allergen specificity. We also provide evidence that lymphocyte-activation gene 3 (Lag3) is a key molecule that contributes to MHC-II antigen recognition and trogocytosis and thus affects the antigen-specific immune regulation of DNT cells.

## Results

### OVA DNTs prevented OVA-induced allergic airway inflammation

**Fig. 1:**
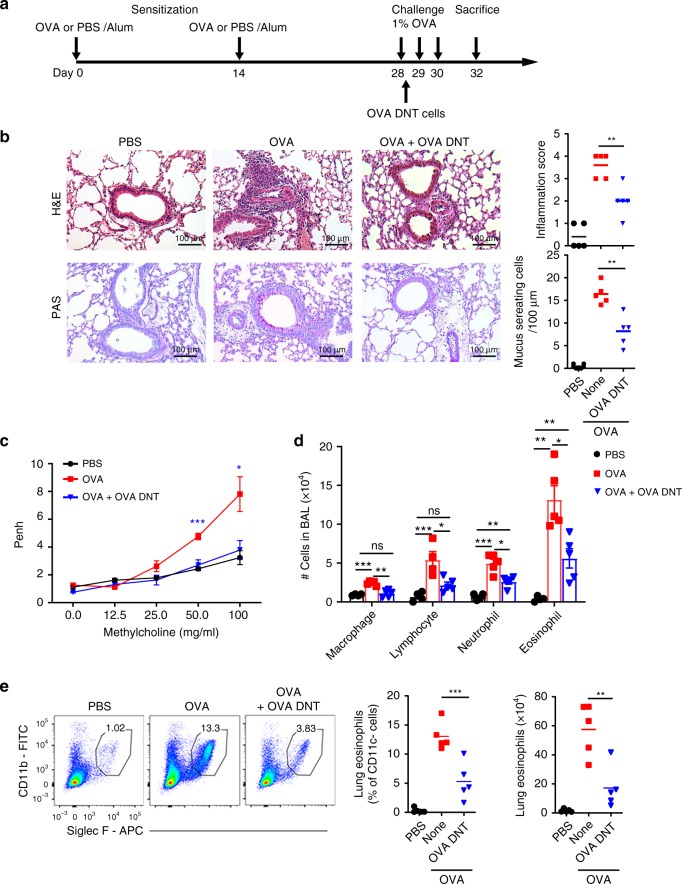
OVA DNTs suppressed OVA-induced airway inflammation. **a** Schematic representation of the experimental procedure. Mice were sensitized with two intraperitoneal injections of ovalbumin (OVA) or PBS in an alum adjuvant at days 0 and 14. The mice received 2 × 10^6^ OVA-primed DNT cells (OVA DNTs) by intravenous adoptive transfer after the first 1% OVA aerosol challenge on day 28. The mice were challenged daily for the next two days and sacrificed 48 h after the last aerosol challenge. **b** Lung sections were stained with H&E and PAS to measure the numbers of infiltrated inflammatory cells and mucus-secreting cells. (Scale bars, 100 µm). **c** The airway hyperreactivity index and the Penh values were investigated 24 h after the last challenge. **d** After the mice were sacrificed, the bronchoalveolar lavage (BAL) fluid was collected and stained with Diff-Quik stain. Cells from the BAL fluid were counted and classified as macrophages, lymphocytes, neutrophils or eosinophils. **e** The percentage and numbers of eosinophils (Siglec F^+^CD11b^+^CD11c^-^) in the lung were assessed by flow cytometry. The results are representative of 4–5 experiments with similar results. Data are shown as the mean ± SEM; *n* = 5 mice per group. One-way ANOVA was used to calculate significance. *P < 0.05; **P < 0.01; ***P < 0.001. The source data are provided as a source data file

To investigate the therapeutic effects of OVA-primed DNT cells (OVA DNTs) in an OVA-induced allergic airway disease model, we adoptively transferred 2 × 10^6^ OVA DNTs into OVA-sensitized mice intravenously after an initial challenge with 1% OVA (Fig. 1a).

As illustrated in Fig. 1b, mice treated with OVA DNTs exhibited significantly reduced inflammatory cell infiltration of the lungs and mucus hypersecretion compared with nontreated mice. To investigate whether lung function was improved after the OVA DNT injection, we measured the Penh index of the mice after DNT cell treatment and OVA challenges. When exposed to high concentrations of methacholine (50–100 mg/ml), the Penh index was significantly lower in the OVA DNT-treated mice compared with the control mice (*P* < 0.001, Fig. 1c).

Significantly decreased numbers of macrophages, lymphocytes, neutrophils, and eosinophils in bronchoalveolar lavage (BAL) fluid were found in OVA DNT-treated mice (Fig. 1d). Flow cytometry analysis also showed a decrease in the proportion and number of lung-infiltrating eosinophils in OVA DNT-treated mice (*P* < 0.001, Fig. 1e, Supplementary Fig. 1A). Furthermore, the proportion of lung macrophages was significantly decreased after OVA DNT treatment (*P* < 0.05, Supplementary Fig. 2A), while the proportions of lung lymphocytes and neutrophils were not changed (Supplementary Fig. 2B and 1C). These results demonstrated that DNT cells could restrict airway inflammation and ameliorate OVA-induced allergic asthma.

### OVA DNTs infiltrated lungs and inhibited cytokine production

**Fig. 2:**
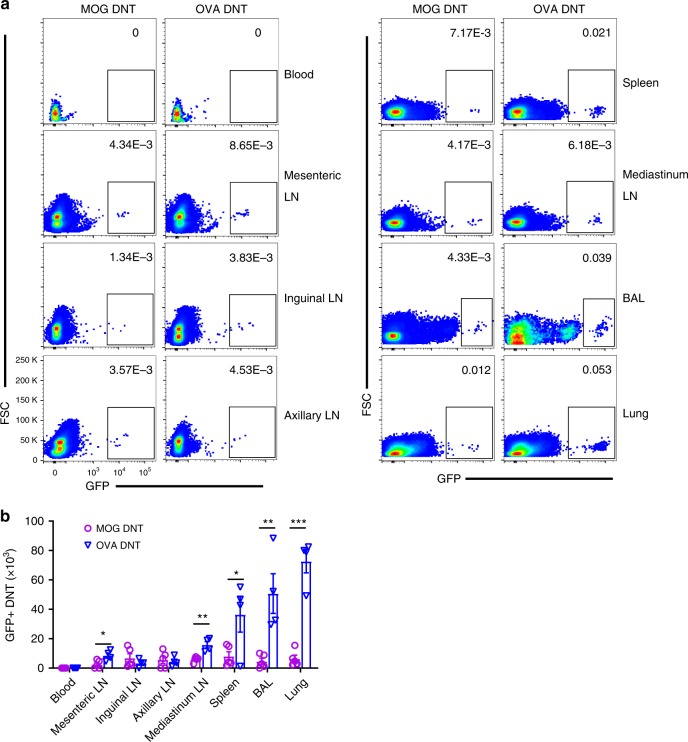
OVA DNTs homed in to the lung to exert a protective effect. GFP^+^CD4^+^ T cells were converted to OVA or MOG DNTs via stimulation with the OVA_323–339_ or MOG_35–55_ peptide. GFP^+^ DNTs were used to treat OVA-induced asthma. **a** The percentages and **b** numbers of GFP^+^ cells in terms of the total cells in the lymphoid tissues were assessed by flow cytometry. Data are shown as the mean ± SEM; *n* = 4 mice per group. Student’s *t-*test was used to calculate significance. *P < 0.05; **P < 0.01; ***P < 0.001. Source data are provided as a source data file

To trace the migration of OVA DNT cells in vivo, we generated MOG- or OVA-primed DNT cells from GFP^+^CD4^+^ T cells. We intravenously injected the same number of GFP^+^ MOG or OVA DNT cells into mice with OVA-induced asthma and measured the accumulation of GFP^+^ DNT cells in different tissues. Compared with MOG DNT cells, OVA DNTs were mainly accumulated in the lungs, BAL, spleen and mediastinal lymph node (mLN) of mice 48 h after the last OVA challenge (Fig. 2a, b). These data suggested that DNT cells could migrate to lung/lung-related tissues to exert antigen-specific protection. Because mice were sensitized by intraperitoneal injections of OVA, it is reasonable that the mesenteric lymph node (mesenteric LN) showed an increased percentage of OVA DNT cells but not MOG DNT cells.

**Fig. 3:**
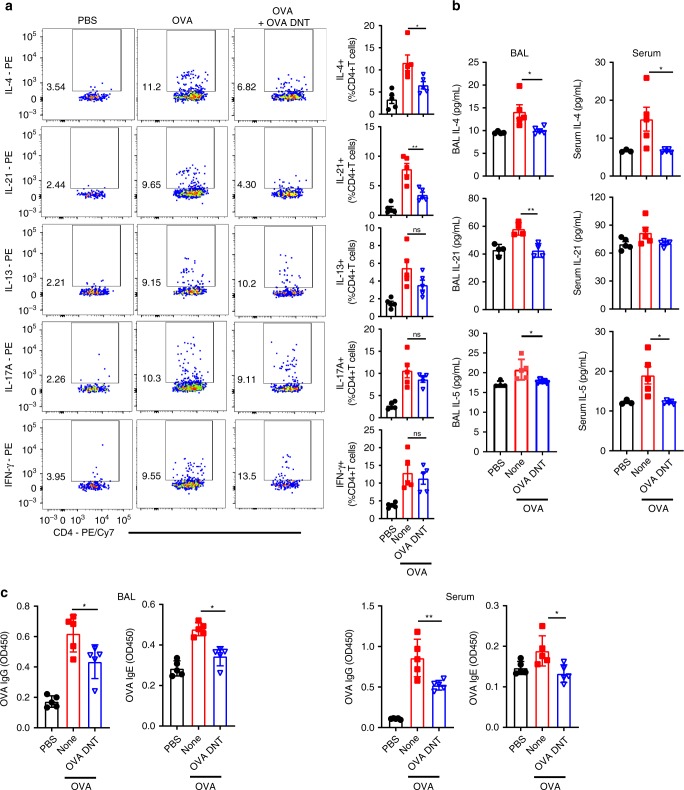
OVA DNTs suppressed cytokine and OVA-specific Ig production. OVA-sensitized mice were treated with an intravenous transfer of OVA DNTs after the first OVA challenge. The mice were challenged daily for the next two days and sacrificed 48 h after the last aerosol challenge. **a** Inflammatory cytokine-secreting lung CD4^+^ T cells were measured by flow cytometry. **b** The BALF and serum cytokine levels were assessed by ELISA. **c** The levels of OVA-specific BALF and serum IgG/IgE were assessed by ELISA. Data are shown as the mean ± SEM; *n* = 4–5 mice per group. One-way ANOVA was used to calculate significance. *P < 0.05; **P < 0.01. Source data are provided as a source data file

To determine whether OVA DNT cells could inhibit cytokine secretion by inflammatory CD4^+^ T cells, we investigated cytokine-secreting CD4^+^ T cells in the lungs after OVA DNT treatment (Fig. 3a). OVA DNT-treated mice showed significantly decreased numbers of IL-4- and IL-21-secreting lung CD4^+^ T cells compared to untreated mice. Meanwhile, we did not find significant differences in IL-13, IL-17 or IFN-γ secretion by CD4^+^ T cells between control and OVA DNT-treated mice. Prominent decreases in serum IL-4 and IL-5 levels and BAL fluid IL-4, IL-5 and IL-21 levels in OVA DNT-treated mice were also revealed (Fig. 3b). Moreover, OVA DNT treatment also markedly decreased the OVA-specific IgG and IgE concentrations in serum and BAL fluid (Fig. 3c).

Overall, these observations indicated that OVA DNTs selectively homed in to lung/lung-related tissues and suppressed IL-4, IL-21, and OVA-specific antibody production in an OVA-induced asthma model.

### DNT treatment reduced the number of Tfh cells and CD11b^+^ DCs

**Fig. 4:**
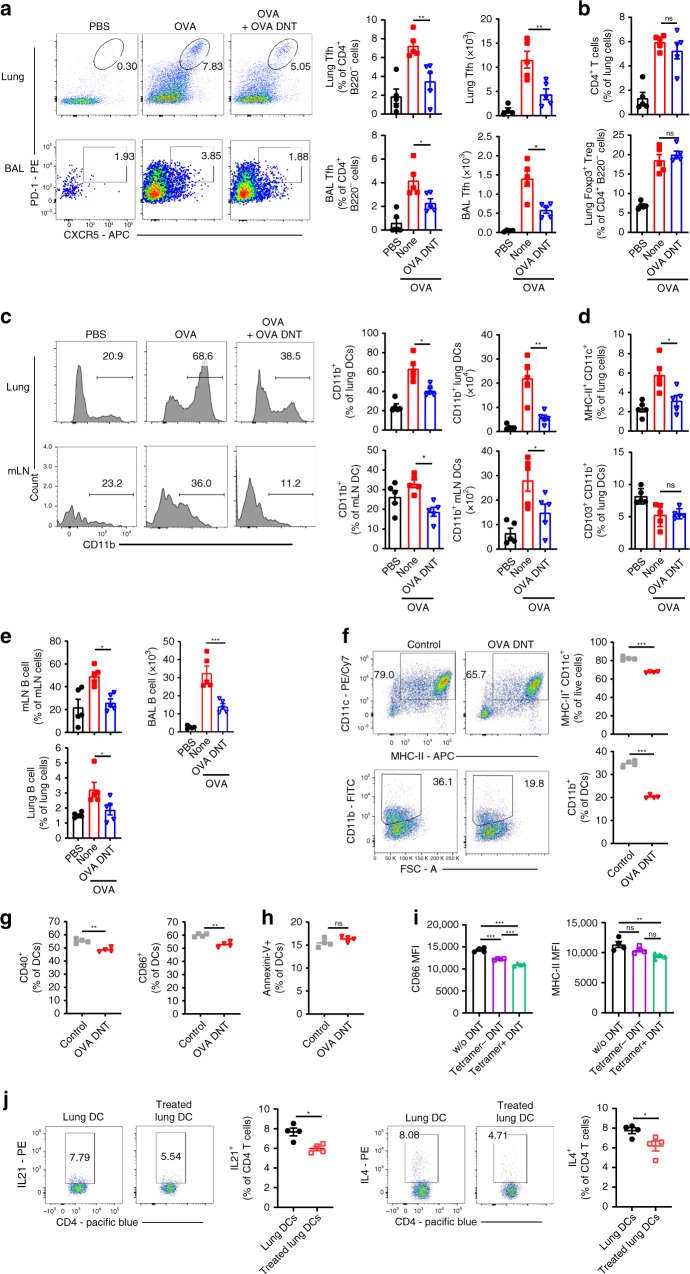
OVA DNT treatment selectively inhibited Tfh cells and CD11b^+^ DCs. OVA-sensitized mice were treated with an intravenous transfer of OVA DNTs after the first OVA challenge. The mice were challenged daily for the next two days and sacrificed 48 h after the last aerosol challenge. **a** The lung and BALF Tfh cell (CD4^+^B220^-^CXCR5^+^PD-1^+^), **b** CD4^+^ T cell (CD4^+^B220^-^) and Treg cell (CD4^+^B220^-^Foxp3^+^) proportions were measured by flow cytometry. **c** The lung and mLN CD11b^+^ DC (CD11c^+^MHC-II^+^CD11b^+^), **d** DC (CD11c^+^MHC-9II^+^) and CD103^+^ DC (CD11c^+^MHC-II^+^CD103^+^CD11b^-^) proportions were measured by flow cytometry. **e** The proportions of B cells (B220^+^CD4^-^) in the mLN (mediastinum lymph node), BALF and lungs were measured by flow cytometry. OVA-stimulated bone marrow cells were cocultured with OVA DNTs and stimulated with GM-CSF (20 ng/ml) for 3 days to test the direct effect of OVA DNTs on OVA DC proliferation and differentiation. **f** The proportions of bone marrow-derived DCs and CD11b+ DCs were measured by flow cytometry. The direct effects of OVA DNTs on **g** costimulatory molecule expression and **h** apoptosis in DCs were measured by flow cytometry. **i** OVA tetramer^+^ and tetramer^-^ DNT cells were sorted by flow cytometry from OVA-primed DNT cells. Tetramer^+^ or tetramer^−^ DNT cells were cocultured with lung DCs from allergic asthma mice for 3 days. The MFIs of CD86 and MHC-II in DCs were measured by flow cytometry. **j** Lung DCs (2.5 × 10^4^) from OVA DNT cell-treated or -untreated asthma mice were cocultured with 1 × 10^5^ naive CD4+ T cells for 3 days. IL21- and IL4-secreting CD4^+^ T cells were measured by flow cytometry. Data are shown as the mean ± SEM; *n* = 4–5 mice per group. One-way ANOVA and Student’s *t*-test were used to calculate significance. *P < 0.05; **P < 0.01; ***P < 0.001. Source data are provided as a source data file

Tfh cells are specialized providers of T cell helper cells for IL-4, IL-21, and antibody production in allergic airway diseases^8,18^. We detected significant decreases in the frequency and total numbers of lung and BAL Tfh cells after OVA DNT treatment (Fig. 4a, Supplementary Fig. 1B). Meanwhile, the changes in the proportions of lung CD4^+^ T cells and Foxp3^+^ Treg cells were not statistically significant (Fig. 4b, Supplementary Fig. 1B). As powerful antigen-presenting cells, CD11b^+^ proinflammatory DCs are responsible for Tfh cell-dependent antibody responses^19^. Our data showed that the frequencies and total numbers of lung and mLN CD11b^+^ DCs were decreased significantly after OVA DNT treatment (Fig. 4c, Supplementary Fig. 1C). Additionally, the proportion of the total CD11c^+^MHC-II^+^ DC population was also decreased after OVA DNT treatment. However, we observed no significant change in the anti-inflammatory CD103^+^ DC population (Fig. 4d). The number of B cells, which produce antibodies, was also decreased significantly in the lungs, mLN and BAL fluid (Fig. 4e, Supplementary Fig. 1B).

To investigate the impact of OVA DNTs on DCs, we assessed DCs in cocultures with OVA DNTs for 3 days in vitro (Fig. 4f–h). As shown in Fig. 4f, the proportions of total CD11b^+^ DCs and MHC-II^+^CD11C^+^ DCs were found to be decreased when they were cocultured with OVA DNTs. Additionally, OVA DNTs also inhibited the expression of the costimulatory molecules CD40 and CD86 by DCs (Fig. 4g). However, we found no significant change in the proportion of apoptotic DCs (Fig. 4h).

Furthermore, we stained OVA-primed DNTs with OVA-specific MHC class II tetramers (I-A^b^ OVA_323–339_ tetramers). Then, we cocultured OVA tetramer^+^ or tetramer^-^ OVA DNTs with lung DCs from OVA-induced allergic mice in vitro for 3 days. As shown in Fig. 4i, both the tetramer^+^ and tetramer^−^ DNTs significantly decreased CD86 expression in DCs, and CD86 expression was markedly decreased when the DCs were cocultured with tetramer^+^ DNTs. Moreover, tetramer^+^ DNT cells but not tetramer^−^ DNT cells could also suppress MHC-II expression by DCs. These results indicated that OVA DNTs selectively inhibited lung DCs, and this inhibition might have been antigen-specific.

To verify whether OVA DNT cell-treated DCs could suppress Tfh and Th2 cell differentiation, we obtained lung DCs from mice treated or untreated with OVA DNT cells. Then, these DCs were cocultured with naive CD4^+^ T cells for 5 days. As shown in Fig. 4j, the OVA DNT cell-treated lung DCs induced significantly lower levels of IL-21 and IL-4 production compared with DCs isolated from untreated mice.

Overall, these data suggested that OVA DNTs selectively reduced the proportion of lung DCs and inhibited their maturation, which contributed to decreased Tfh and Th2 cell differentiation.

### Allergen-specific inhibition of inflammation by OVA DNTs

**Fig. 5:**
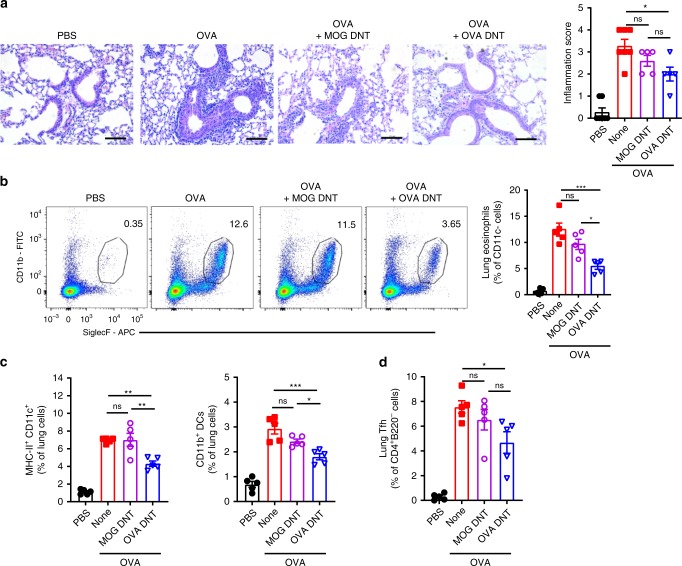
OVA DNTs specifically protected mice against OVA-induced airway inflammation. Mice received MOG DNTs or OVA DNTs by intravenous adoptive transfer to treat OVA-induced airway inflammation. **a** Lung sections were stained with H&E to measure the accumulation of infiltrating inflammatory cells. (Scale bars, 100 μm). **b** Eosinophils (Siglec F^+^CD11b^+^CD11c^−^) in the lung were assessed by flow cytometry. **c** Lung DCs (CD11b^+^CD11c^+^MHC-II^+^) and **d** Tfh cells (CD4^+^B220^-^CXCR5^+^PD-1^+^) were assessed by flow cytometry. The results are representative of two experiments with similar results. Data are shown as the mean ± SEM; *n* = 5 mice per group. One-way ANOVA was used to calculate significance. *P < 0.05; **P < 0.01; ***P < 0.001. Source data are provided as a source data file

Based on the observation that OVA DNTs efficiently inhibited OVA-induced allergic airway inflammation, we questioned whether OVA DNTs acquired allergen specificity after OVA_323–339_ peptide stimulation. CD4^+^ T cells were converted to DNT cells with either OVA_323–339_ peptide or an unrelated peptide (MOG_35–55_). No differences in the expression of CD11b, CD11c, MHCII, or CXCR5 were found between OVA DNTs and MOG DNT cells (Supplementary Fig. 3). C57BL/6 mice were sensitized, challenged with OVA and treated with OVA DNTs or MOG-primed DNT cells (MOG DNTs) as previously described. Intriguingly, the MOG DNTs failed to inhibit OVA-induced allergic airway inflammation and lung eosinophil accumulation (Fig. 5a, b). Similarly, we found no statistically significant decreases in the accumulation of DCs, CD11b^+^ DCs and Tfh cells after MOG DNT treatment (Fig. 5c, d).

To further confirm the antigen-specific suppression of DNT cells in allergic asthma, we induced allergic asthma with HDM (house dust mite) extract in BALB/c mice (Supplementary Fig. 4A). HDM or OVA-primed DNT cells were adoptively transferred to mice with HDM-induced allergic asthma. Similar to the previous results, HDM DNT cells, rather than OVA DNT cells, significantly ameliorated inflammatory cell infiltration (Supplementary Fig. 4B) and reduced the accumulation of eosinophils (Supplementary Fig. 4C), Tfh cells (Supplementary Fig. 4D) and CD11b+ DCs (Supplementary Fig. 4E).

These results indicated that DNT cells suppressed allergic airway inflammation while maintaining allergen specificity.

### Lag3 depletion reduced suppressor activity of OVA DNTs

**Fig. 6:**
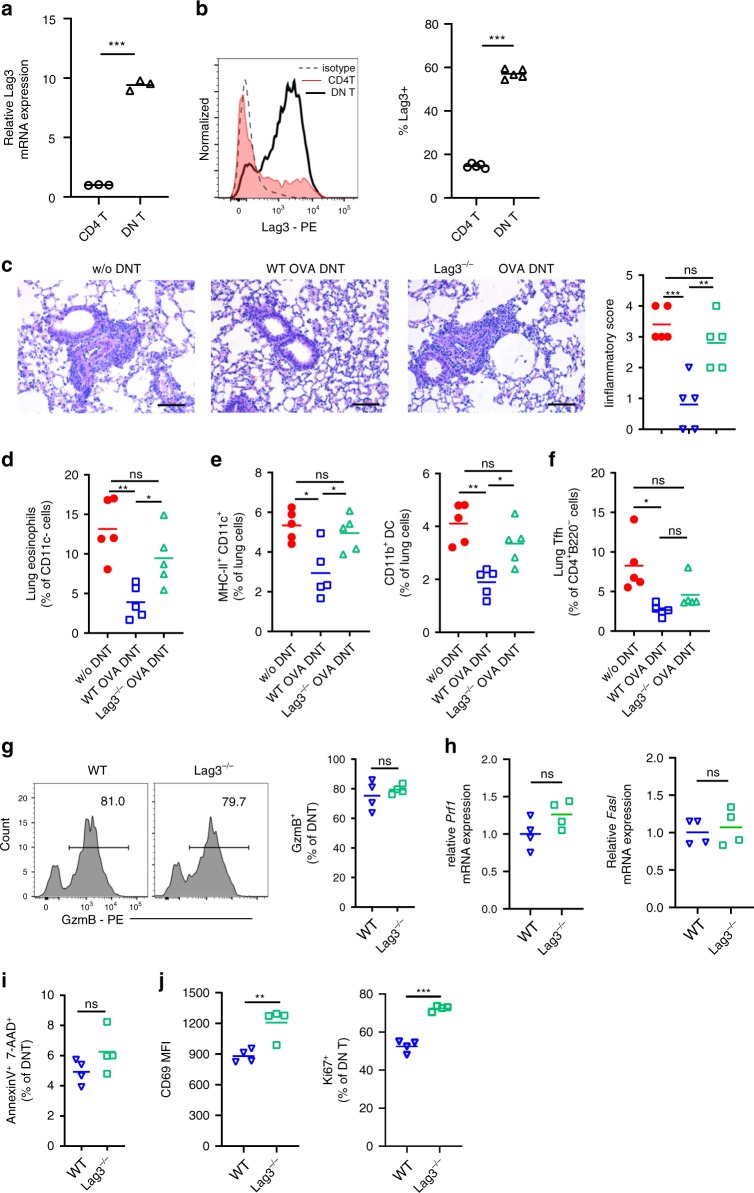
Lag3 knockout reduced the antigen-specific suppression of OVA DNTs. Relative *Lag3* mRNA expression in OVA DNTs and CD4^+^ T cells was measured by **a** real-time PCR and **b** flow cytometry. Mice received WT OVA DNTs or *Lag3*^*−/−*^ OVA DNTs by intravenous adoptive transfer to treat OVA-induced airway inflammation. **c** Lung sections were stained with H&E to measure the accumulation of infiltrating inflammatory cells (Scale bars, 100 μm). **d** Lung eosinophils (CD11b^+^Siglec F^+^CD11c^-^), **e** DCs (CD11c^+^MHC-II^+^), CD11b^+^ DCs (CD11b^+^CD11c^+^MHC-II^+^) and **f** Tfh cells (CD4^+^B220^-^CXCR5^+^PD-1^+^) were assessed by flow cytometry. **g** GzmB expression in WT DNTs and *Lag3*^*−/−*^ DNTs were measured by flow cytometry. **h** Relative *Prf1* and *Fasl* mRNA expression levels in WT DNTs and *Lag3*^*−/−*^ DNTs were measured by real-time PCR. **i** The apoptosis of DNT cells was detected by flow cytometry. **j** The expression of CD69 and Ki67 were detected by flow cytometry. Data are shown as the mean ± SEM; *n* = 4–5 mice per group. One-way ANOVA and Student’s *t*-test were used to calculate significance. *P < 0.05; **P < 0.01; ***P < 0.001. Source data are provided as a source data file

CD4 molecules are essential coreceptors of T cell receptors that contribute to the recognition of peptide antigen presented by MHC-II molecules of antigen-presenting cells. The way in which DNT cells retain antigen specificity and recognize OVA peptides presented by dendritic cells without CD4 expression is still unknown. Lag3 has been reported to be a molecule that can bind to MHC-II with higher affinity than CD4^20^. We, therefore, measured Lag3 expression by DNT cells. Compared with CD4^+^ T cells, DNT cells showed significantly upregulated *Lag*3 expression according to real-time PCR results (Fig. 6a). The markedly increased expression of Lag3 was also confirmed by flow cytometry (Fig. 6b).

To reveal whether Lag3 expression by DNT cells contributes to antigen specificity in DNT cells, we compared the suppressive function of Lag3-deficient DNT cells with that of WT DNT cells in vivo. CD4^+^CD25^-^ T cells from WT or *Lag3*^*−/−*^ mice were converted to OVA DNTs. As shown in Fig. 6c, the adoptive transfer of *Lag3*^*−/−*^ OVA DNTs failed to ameliorate OVA-induced airway inflammation. Additionally, the percentages of eosinophils, DCs and CD11b^+^ DCs showed no significant differences between the *Lag3*^*−/−*^ OVA DNT-treated group and the control groups (Fig. 6d, e). Given the intimate link between DCs and Tfh cells, we also observed no significant change between the Tfh cell population of the *Lag3*^*−/−*^ OVA DNT-treated group and that of the control groups (Fig. 6f).

DNT cells exert control over immune responses mainly through the perforin/granzyme and Fas/Fas L pathways^13,15,21^. To investigate whether the weakening of the immunosuppressive activity of the *Lag3*^*−/−*^ OVA DNTs was associated with the downregulation of these pathways, we assessed suppressive gene expression in DNT cells. As shown in Fig. 6g, no significant differences in granzyme B expression were observed between WT and *Lag3*^*−/−*^ OVA DNTs by flow cytometry. The mRNA expression levels of *Prf1* and *Fasl* were also similar in WT and *Lag3*^*−/−*^ OVA DNTs (Fig. 6h). The proportion of apoptotic DNT cells increased slightly among the *Lag3*^*−/−*^ cells, but the difference was not significant (Fig. 6i). Intriguingly, similar to CD4^+^ T cells^22^, *Lag3*^*−/−*^ DNT cells expressed significantly increased levels of the cell activation marker CD69 and the proliferation marker Ki67 than WT DNT cells (Fig. 6j).

Overall, Lag3 depletion reduced the antigen-specific suppression of OVA DNTs, and this reduction in suppression was not related to DNT cell activation, apoptosis, or perforin, granzyme or Fas L expression.

### Lag3 contributed to antigen recognition by DNT cells

**Fig. 7:**
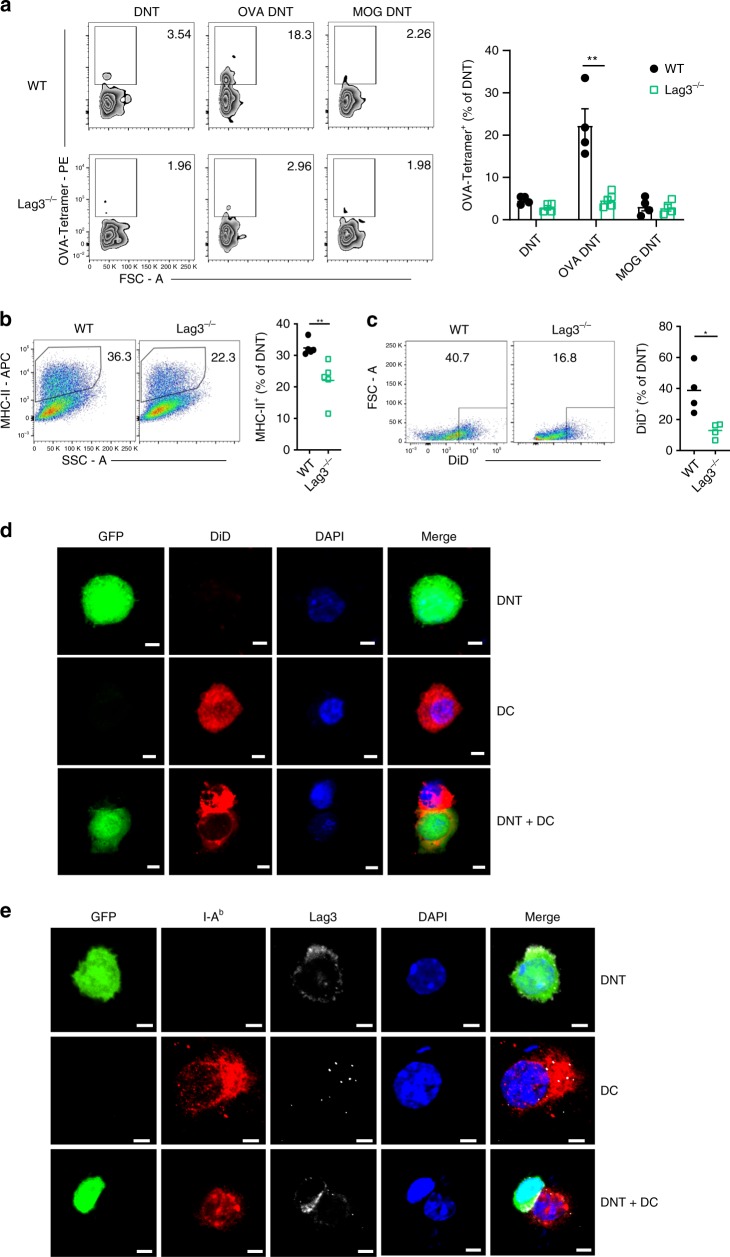
The antigen-specific suppression of OVA DNTs was Lag3-dependent. **a** OVA-specific DNT cells were assessed by OVA-tetramer-PE staining. WT or *Lag3*^*−/−*^ CFSE-labeled DNT cells were incubated with DiD-labeled DCs for 24 h. **b** MHC-II molecule and **c** DiD capture by DNT cells after coculture were measured by flow cytometry. The results are representative of two experiments with similar results. **d** GFP^+^ DNT cells were analyzed by confocal fluorescence microscopy after being incubated with DiD-labeled lung DCs for 24 h (Scale bars, 5 μm). **e** I-A^b^ expression on DCs and Lag3 expression on GFP^+^ DNT cells were analyzed by confocal fluorescence microscopy. (Scale bars, 5 μm). Data are shown as the mean ± SEM; *n* = 4–5 mice per group. Student’s *t*-test was used to calculate significance. *P < 0.05; **P < 0.01. Source data are provided as a source data file

To investigate the impact of Lag3 on antigen-specific recognition by DNT cells, we assessed the WT and *Lag3*^*−/−*^ OVA DNTs by staining them with OVA-specific MHC class II tetramers (I-A^b^ OVA_323–339_ tetramers) (Fig. 7a). A significantly higher proportion of I-A^b^ OVA_323–339_ tetramer-positive cells was observed in the OVA DNT cells compared with either the OVA-primed *Lag3*^*−/−*^ DNT cells or the MOG-stimulated WT DNT cells. In contrast, the proportion of OVA tetramer-positive cells in the *Lag3*^*−/−*^ DNT cells primed with the OVA_323-339_ peptide was not significantly different from that in either the WT or Lag 3-deficient DNT cells that were stimulated with MOG peptide (Fig. 7a). To clarify whether Lag3 is also important for antigen-specific recognition by natural DNT cells, naive natural DNT cells from WT or *Lag3*^*−/−*^ mice were cocultured with C57BL/6J mDCs, 50 ng/ml rmIL-2 and 1 μg/ml OVA_329–339_ for 5 days. The activated and freshly isolated naive WT or *Lag3*^*−/−*^ DNT cells were stained with OVA-specific MHC class II tetramers (I-A^b^ OVA_323–339_ tetramers). As shown in Supplementary Fig. 5, although the average OVA tetramer-positive cell percentage was lower in the natural DNT cells than the CD4 T cells that were stimulated to become DNT cells, a significantly higher proportion of I-A^b^ OVA_323-339_ tetramer-positive cells was still observed in the OVA-primed natural WT DNT population compared with either the OVA-primed *Lag3*^*−/−*^ natural DNT cell or naive DNT cell population. These results indicated that Lag3 was also involved in the antigen recognition of natural DNT cells.

Recent evidence demonstrated that trogocytosis is important for antigen acquisition by DNT cells^23^. As shown in Fig. 7b, c, lung DCs from asthmatic mice were stained with the molecular probe DiD, which is highly fluorescent when incorporated into membranes^24^, and cocultured with DNT cells for 24 h. MHC-II molecule expression and DiD staining were markedly decreased in *Lag3*^*−/−*^ DNT cells than in WT DNT cells. To observe the trogocytosis process directly, we cocultured GFP^+^ DNT cells and lung DCs labeled with DiD. Confocal microscopy revealed that DiD-labeled fragments of DC plasma membranes were translocated into DNT cells (Fig. 7d). Moreover, the DCs and GFP^+^ DNT cells were also stained for surface Lag3 and MHC-II, respectively. Interestingly, we observed that Lag3 was mainly enriched on the surfaces where DNT cells had had direct contact with DCs (Fig. 7e).

Overall, these data demonstrated that Lag3 was important for the interaction between DNT cells and antigen-presenting cells. DNT cells could acquire MHC-II molecules from dendritic cells to obtain antigen specificity with the assistance of Lag3.

## Discussion

Current treatments for allergic and autoimmune diseases depend on nonspecific immune suppression and often lead to adverse reactions or other conditions in patients. Innovative strategies that target allergen-induced specific immune responses will provide the benefit of preventing unwanted adverse effects or deaths and improve the quality of life of patients.

DNT cells are a unique type of regulatory T cells and are essential for maintaining immune system homeostasis^13,14^. We previously identified the differentiation pathway that converts CD4^+^ T cells into DNT cells^15,21^. CD4^+^ T cell-converted DNT cells potently suppressed vigorous allo- and autoimmune responses, prolonged islet and skin allograft survival, and prevented and cured autoimmune type 1 diabetes^15–17^.

In this study, to develop a cellular therapy for allergic asthma, we converted naive CD4^+^ T cells to OVA-specific DNT cells ex vivo. The adoptively transferred OVA-primed DNT cells mainly accumulated in the lungs, BALF and spleen to significantly inhibit OVA-induced airway inflammation and suppress mucus hypersecretion, bronchial hyperreactivity and the infiltration of eosinophils and lymphocytes. The protection from OVA-induced allergic airway inflammation mediated by DNT cells was mediated by the inhibition of Tfh cells and CD11b^+^ DCs and the reduced secretion of IL-21, IL-4, and OVA-specific antibodies.

Tfh cells are a subset of T helper cells that provide specialized help to B cells and facilitate antibody production^25^. Additionally, Tfh cells are responsible for the initiation of type 2 allergic asthma^8,9,26^. Our study shows that DNT cell treatment significantly reduced the proportion of Tfh cells and decreased IL-21 secretion in OVA-induced allergic airway disease, which demonstrated the direct effects of DNT cells on ameliorating allergic asthma. Tfh cell priming requires costimulatory signals from DCs in lymphoid tissues, and antigen presentation by DCs is essential for optimal antigen-specific Tfh cell development^25^. The key role of CD11b^+^ DCs in initiating allergic responses has been extensively demonstrated^27,28^. Additionally, CD11b^-^ DCs, especially CD103^+^ DCs, seem to restrain allergic airway inflammation^29,30^. Our data herein show that OVA DNT treatment induced a selective reduction in the proportion of CD11b^+^ DCs, which are the predominant Tfh-promoting DC subset. We did not observe any appreciable changes in regulatory CD103^+^ DCs or Foxp3^+^ Treg cells after OVA DNT treatment. In vitro coculture of bone marrow-derived DCs and DNT cells revealed the significant suppressive effect of DNT cells on the proliferation, differentiation and costimulatory molecule expression of CD11b^+^ DCs. OVA DNT cell-treated lung DCs induced significantly reduced IL-21 and IL-4 production compared with DCs isolated from untreated mice. These data suggested that DNT cells have direct suppressive effects on CD11b^+^ DCs, which repress further Tfh cell activation and cytokine secretion.

Previous studies demonstrated that both murine and human DNT cells suppress allogeneic immune responses and autoimmune responses in an Ag-specific fashion^11,12,15,16^. T cells have been shown to acquire MHC class I and class II molecules from antigen-presenting cells^31,32^. Ford et al. showed that naive DNT cells can also acquire alloantigens through trogocytosis in vivo^23^. In an allogeneic hematopoietic stem cell transplantation study, DNT cells acted as MHC class I/peptide reactive T cells and contributed to antiviral immune responses; these conclusions were supported by an HLA-A*24:02 EBV tetramer test and the observation of EBV (BZLF1)-specific cytokine secretion in response to certain peptides^33^. Leishmania-reactive DNT cells were reported to be MHC-II restricted, as anti MHC-II antibodies blocked the proliferation and IFN-γ production of both CD4^+^ and DNT cells in a dose-dependent manner. In addition, Leishmania-infected BMDCs from MHC-II KO mice failed to induce proliferation and IFN-γ production in DN and CD4^+^ T cells. In contrast, proliferation and IFN-γ production in DNT cells were minimally affected following coculture with infected BMDCs from CD1d KO mice, confirming that DNT cells are mostly MHC-II restricted^34^. However, as DNT cells lack the expression of the MHC-II coreceptor CD4, the recognition of MHC-II and maintenance of antigen specificity by DNT cells remain unclear.

In our study, compared with CD4^+^ T cells, DNT cells exhibited elevated Lag3 expression. Lag3 is a transmembrane protein that belongs to the immunoglobulin superfamily. A comparative peptide analysis of Lag3 and CD4 showed the close relationship between the two molecules^35^. Huard et al. demonstrated that compared with the corresponding CD4 molecule, both human and mouse Lag3 show 100-fold higher avidity towards MHC-II^20^. Intriguingly, in our study, a significantly higher proportion of I-A^b^ OVA_323–339_ tetramer-positive cells was observed among OVA-primed DNT cells compared with *Lag3*^*−/−*^ DNT cells. Lag3-deficient DNT cells showed weak MHC-II recognition and therapeutic effects on OVA-induced asthma. Furthermore, we observed Lag3 accumulation at the surfaces of contact between DNT cells and DCs. These results indicated the important role of Lag3 in the interaction of DNT cells and DCs. Trogocytosis is a cell–cell contact-dependent membrane transfer process that usually occurs between lymphocytes and antigen-presenting cells^36^. In this study, we also identified the intercellular transfer between MHC-II and the plasma membrane mediated by the Lag3 molecule between DNT cells and DCs. Thus, our study revealed that DNT cells exhibited trogocytosis and attained antigen specificity with the assistance of Lag3.

Lag3 is one of the putative markers of mouse and human T regulatory type 1 cells (Tr1) cells^37^. Tr1 cells preferentially produce IL-10 and exert immunosuppressive effects. The adoptive transfer of Lag3^+^CD49b^+^CD4^+^ Tr1 cells ameliorates allergic asthma^10^. However, whether Lag3 regulates the immunosuppressive function of Tr1 cells is unclear. Lag3 was also reported to be essential for Foxp3^+^ Treg functioning, as Lag3^–/–^ Tregs exhibit reduced suppressive activity^38^. It was reported that Lag3 intrinsically limited Treg proliferation and functioning at sites of inflammation in an autoimmune diabetes model^39^. The adoptive transfer of Lag3-deficient Tregs was unable to attenuate allergic inflammation, demonstrating the critical role of the IL-27/Lag3 axis in mediating Treg control of allergic inflammation^40^. Unlike Tr1 cells and Foxp3^+^ Tregs, which both highly express Lag3, DNT cells do not produce IL-10 ^21^ and show no Foxp3^15,21^ expression. Lag3 expression on DNT cells contributes to MHC-II antigen recognition and thus affects antigen-specific immune regulation by DNT cells.

In conclusion, ex vivo-generated, CD4 T cell-converted, allergen peptide-primed DNT cells exerted potent antigen-specific immune regulatory effects in allergen-induced mouse asthma models. Elevated Lag3 expression and trogocytosis contributed to MHC-II antigen recognition by DNT cells. These data support the concept and the feasibility of potentially utilizing this cell-based allergen-specific therapeutic approach for the clinical treatment of allergy and asthma.

## Methods

### Mice

Wild type (WT) C57BL/6J, C57BL/6J-GFP, BALB/c, and *Lag3*^*−/−*^ mice were purchased from Jackson Laboratory. The animals were housed and bred under specific pathogen-free conditions in a temperature-controlled environment under 12 h light/dark cycles at Beijing Friendship Hospital. All procedures were performed in accordance with the guidelines of the Institutional Animal Care and Ethics Committee at Beijing Friendship Hospital.

### Conversion of DNT cells in vitro and adoptive transfer

The conversion of DNT cells in vitro was performed as previously described^15,16^. Briefly, mature dendritic cells (mDCs) were harvested from lipopolysaccharide-stimulated bone marrow cells derived from C57BL/6J mice and separated according to CD86-positive selection. C57BL/6J or C57BL/6J-GFP CD4^+^ T cells were incubated with C57BL/6J mDCs, 50 ng/ml rmIL-2 (PeproTech, USA) and 1 μg/ml OVA_329–339_ peptide, MOG_35-55_ peptide (Sigma-Aldrich) or HDM extract (Greer) for 7 days. CD3^+^CD4^−^CD8^−^NK1.1^−^ DN cells were sorted using a FACSAriaII sorter (BD Biosciences, USA).

### Asthma model and DNT cell treatment

Six- to eight-week-old male C57BL/6J mice were sensitized by i.p. injections of 20 µg OVA (Sigma-Aldrich) in 50 μl Imject™ alum adjuvant (Thermo Fisher Scientific) in a total volume of 100 μl on days 0 and 14. The control mice received alum adjuvant (PBS) only. Beginning on day 28 after the injections, the mice were exposed to aerosolized 1% OVA (in 0.85% NaCl solution) for 30 min/day for 3 consecutive days. On day 28, the ex vivo-converted OVA-primed DNT cells (2 × 10^6^) were transferred to the mice by tail vein injection after the first inhalation of 1% OVA. In the HDM-induced allergic asthma model, 8-week-old female BALB/c mice were sensitized by the i.p. injection of 100 µg HDM extract (Greer) in 50 μl Imject™ alum adjuvant. The sensitized mice were administered (i.n.) 25 µg of HDM extract intranasally daily for 5 days starting at day 7. Ex vivo-converted HDM-primed DNT cells (2 × 10^6^) were transferred into mice by tail vein injection after the first i.n. challenge.

### Measurement of airway hyperreactivity to methacholine

24 h after the last OVA aerosol challenge, airway hyperresponsiveness to methacholine was determined using noninvasive unrestrained whole body plethysmography (EMKA Technologies). The mice were placed in individual chambers and exposed to nebulized methacholine (0, 12.5, 50, or 100 mg/ml in PBS, 0 mg/ml as baseline) for 2 min followed by a 1 min rest. The enhanced pause (Penh) was then measured for 3 min. The average Penh value were expressed for each methacholine concentration in comparison with the baseline Penh values.

### Tissue processing

The tracheas were cannulated and washed twice with 1 ml of PBS before isolating the lungs. The lungs, lymph nodes (mesenteric, inguinal, axillary and mediastinum) and spleens were processed in RPMI 1640 medium. PBS was injected into the right ventricle to flush the circulating blood cells. The lungs were chopped into small pieces and digested with tissue digestion solution (0.5 mg/ml collagenase IV plus 8 μg/ml DNase I in HBSS containing 5% FBS) for 20 min before passing the tissue through a 70-µm cell strainer (BD Biosciences). Single cells were selected from the Aqua-stained (live) and CD45^+^ cell populations. RBC lysis buffer (Qiagen) was used to lyse the red blood cells. The bronchoalveolar lavage (BAL) fluid was stained with the Diff-Quik stain kit (Solarbio) according to the manufacturer’s instructions.

### Ig and cytokine detection

OVA-specific IgG and IgE levels were determined in the bronchoalveolar lavage (BAL) and serum samples collected at the end of the experiments. Briefly, for OVA-specific IgG and IgE, high-binding plates were coated overnight at 4 °C with 5 μg/ml OVA in carbonate buffer, blocked with 2% milk/PBS, and incubated with 1:1000–1:10,000 serum dilutions. The OVA-specific immunoglobulins were detected with an HRP-conjugated rat anti-mouse IgG antibody (Zsbio). The signal was developed by incubation with a standard TMB solution, and the optical density was read at 450 nm. The total IgE level was quantified with a capture mAb, biotin detection mAb, and streptavidin-HRP from the Mouse IgE ELISA Ready-SET-Go! kit (eBioscience). Readings were also obtained to generate a standard curve prepared with purified mouse IgE.

IL-21 production was quantified in serum and BALF with a mouse IL-21 ELISA kit (eBioscience) according to the manufacturer’s instructions. The standard curve was prepared with purified mouse IL-21. The concentrations of IL-4 and IL-5 were detected with the LEGENDplex Multi-Analyte Flow Assay Kit for Mouse Th Cytokine (BioLegend) according to the manufacturer’s instructions. Analyses were performed with a FACSAriaII (BD Biosciences) and the LEGENDplex software (BioLegend).

### Histological analysis

Serial sections were prepared from formalin-fixed, paraffin-embedded lung tissue. The sections were stained with H&E or with periodic acid-Schiff (PAS) reagent and scanned at ×20 magnification with a DM 2500 (Leica Corporation). Images were prepared using LAS version 4.6.1 software (Leica Application Suite software). The mucus-secreting cells around the airways (mean in 10 × 100 μm fields) were detected using light microscopy. The number of mucus-filled (PAS+) cells/100 μm airway epithelium were enumerated in a blinded manner^41^. The inflammatory infiltrate analysis was scored as follows: absent was scored as 0, 1 denoted ‘rare infiltrate’, 2 denoted ‘mild’ (only in a focal area), 3: denoted ‘moderate’ (<5 cell lines deep) and 4: denoted ‘severe’ (>5 lines of cells deep)^42^. All scores were enumerated in a blinded manner by 2 blinded independent investigators.

### Flow cytometry

Anti-CD11b (ICRF44, dilution 1:200, Cat 101206), anti-CD11c (N418, dilution 1:200, Cat 117318), anti-I-A/I-E (M5/114.15.2, dilution 1:200, Cat 107614), anti-CD8a (53-6.7, dilution 1:500, Cat 100714), anti-CD103 (M290, dilution 1:200, Cat 557495), anti-CD40 (3/23, dilution 1:100, Cat 124611), anti-CD80 (16-10A1, dilution 1:200, Cat 104707), anti-Siglec F (E50-2440, dilution 1:200, Cat 562680) and anti-Ly-6G (RB6-8C5, dilution 1:200, Cat 108408) antibodies were used to identify the DC and granulocyte populations. Anti-CD4 (GK1.5, dilution 1:500, Cat 100428), anti-B220 (RA3-6B2, dilution 1:400, Cat 103206), anti-CXCR5 (SPRCL5, dilution 1:100, Cat 551960), anti-Foxp3 (PCH101, dilution 1:100, Cat 45-4776-42), anti-GzmB (GB11, dilution 1:200, Cat 561142) and anti-PD-1 (29F.1A12, dilution 1:200, Cat 135216) antibodies and Streptavidin-APC (dilution 1:200, Cat 405207) were used to identify the T cell populations. Anti-IL4 (11B11, dilution 1:200, Cat 504103), anti-IL-13 (eBio13A, dilution 1:200, Cat 12-7133-41), anti-IL-17A (TC11-18H10, dilution 1:200, Cat 506903), anti-IL-21 (4A9, dilution 1:200, Cat 131905) and anti-IFN-γ (XMG1.2, dilution 1:200, Cat 505808) antibodies were used to identify cytokine-producing CD4^+^ T cells. Zombie Aqua^TM^ Fixable Viability kits were used to exclude dead cells. The Zombie Aqua^TM^ Fixable Viability kits and fluorochrome-conjugated antibodies were purchased from BioLegend, eBioscience or BD Pharmingen.

For OVA tetramer staining, suspended cells were stained with I-A^b^ OVA_323–339_ tetramer-PE (MBL) at 4 °C for 1 h. The surface marker antibodies were added without washing and incubated for 15 min. The cells were washed once before flow cytometry detection. The data were collected using a FACSAriaII (BD Biosciences) and analyzed with FlowJo software (Tree Star).

### DNT cell and DC coculture

OVA DNT cells were converted from CD4^+^ T cells as described above. CD11c^+^MHC-II^+^ lung DCs were sorted from OVA-induced allergic asthma mice by a FACSAriaII sorter. A total of 4 × 10^4^ DCs were cocultured with 2 × 10^4^ OVA DNTs for 3 days. The proportion and costimulatory molecule expression of DCs were assessed by flow cytometry.

### DiD labeling & confocal microscopy

CD11C^+^MHC-II^+^ lung DCs were sorted by flow cytometry. 5 µg/mL DiD dye (Thermo Fisher Scientific) was added to 1 × 10^6^/mL DCs suspended in serum-free RPMI 1640 medium. The DCs were incubated for 20 min at 37 °C and washed twice with RPMI 1640 medium containing 10% FBS. To investigate the trogocytosis process directly, we cocultured 5 × 10^5^ GFP^+^ OVA DNTs and DiD-labeled DCs for 24 h at a ratio of 1:1 in a 24-well plate. For confocal microscopy, the antibodies used were rabbit anti-mouse Lag3 antibody (Abcam) and mouse-purified anti-mouse I-A^b^ (Biolegend), and DAPI was used as a nuclear stain (Molecular Probes). Donkey anti-rabbit IgG Alexa Fluor 546 and donkey anti-mouse IgG Alexa Fluor 647 (Thermo Fisher Scientific) were used to detect the primary Abs. Confocal analysis was conducted using a confocal laser scanning microscope (FLUOVIEW FV1000, Olympus). The image data were acquired by FV10-ASW 4.2 microscopy software.

### RNA extraction and real-time PCR

Total RNA was extracted with a RNeasy Micro Kit (Qiagen), and the cDNA was reverse transcribed with a Prime Script® RT reagent Kit (Takara). Real-time PCR was performed with the 7500 Fast Real-time System (Applied Biosystems) using SYBR Green Master Mix (Applied Biosystems). The primers used in this study are listed in Supplementary Table 1. The real-time PCR relative values were calculated with the comparative Ct method and were normalized against the expression of the housekeeping gene *β-actin*.

### Statistics

The statistical analyses were performed with GraphPad Prism software (GraphPad Software Inc., USA), and the experimental data are presented as the mean ± standard deviation (SD). One-way ANOVA with a Bonferroni or Tukey posttest was used for multiple comparisons; a 2-tailed, unpaired *t*-test was used for unmatched pairwise sample comparisons (SPSS 23). Significant differences are shown as *P < 0.05, **P < 0.01, and ***P < 0.001.

### Reporting summary

Further information on research design is available in the Nature Research Reporting Summary linked to this article.

## Supplementary information

Supplementary Information

Reporting Summary

Source Data

## Data Availability

The data supporting the findings of this paper are available from the corresponding author upon reasonable request. The source data underlying Figs. 1b–e, 2b, 3a–c, 4a–j, 5a–d, 6a–j, 7a–e, S2, S3, S4B–E and S5 are provided as a source data file.
