# Supplementary material for: Double negative T cells mediate Lag3-dependent antigen-specific protection in allergic asthma
Source: Nat Commun. 2019 Sep 18;10:4246. doi: 10.1038/s41467-019-12243-0 (PMC6751182; doi:10.1038/s41467-019-12243-0)
Supplement: Supplementary file 1 — Supplementary Information [file 41467_2019_12243_MOESM1_ESM.pdf]

Double negative T cells mediate Lag3-dependent antigen-specific protection in allergic asthma

Tian et al.

Supplementary Figure 1

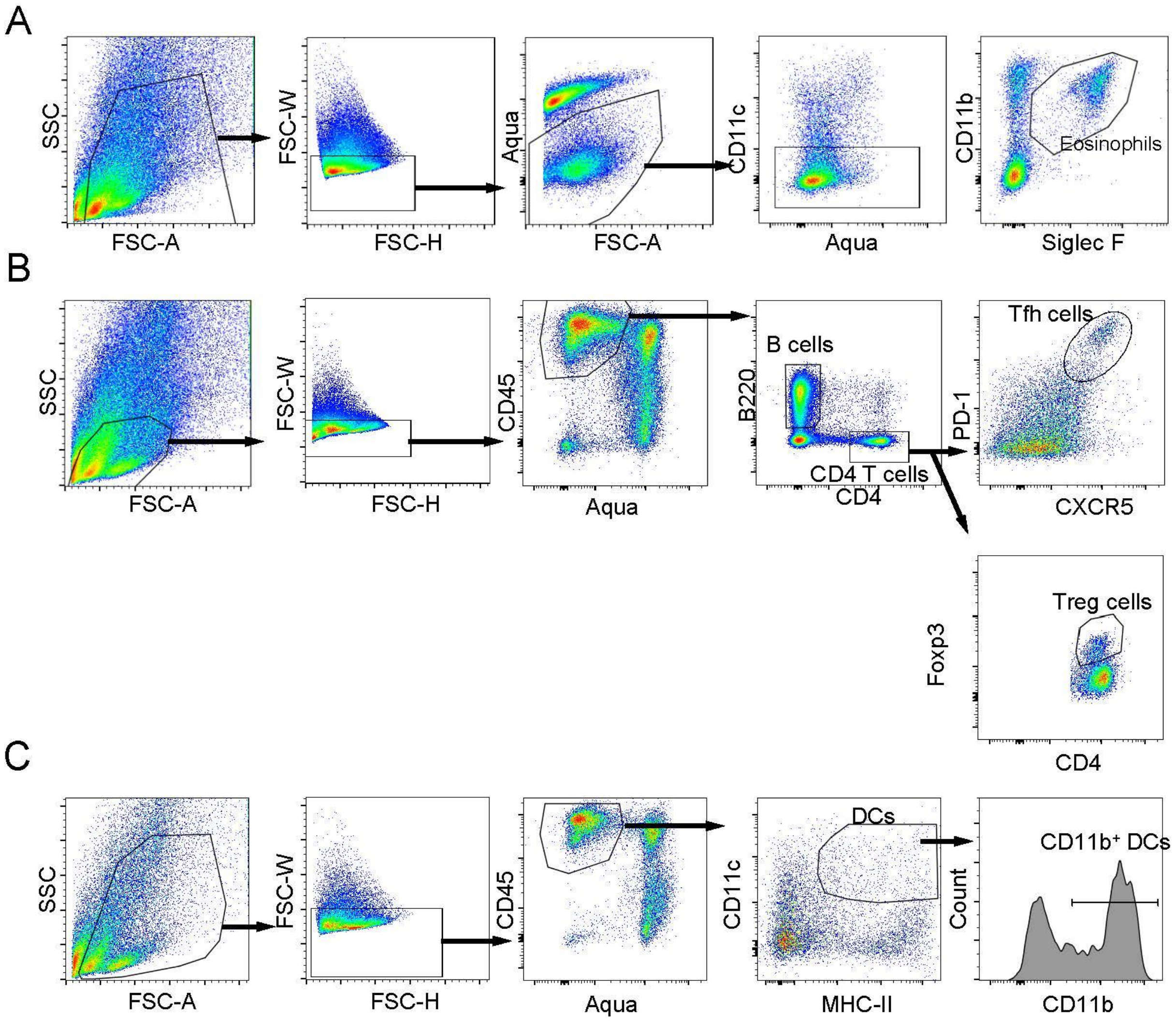

Supplementary Figure 1. Gating strategies of flow cytometry.

(**A**) Gating strategy to detect lung eosinophils (Siglec F$^+$CD11b$^+$CD11c$^-$) presented on Fig. 1E, Fig. 5B and Fig. 6D. (**B**) Gating strategy to detect lung DCs (CD11c$^+$ MHC-II$^+$) and CD11b$^+$ DCs (CD11c$^+$ MHC-II$^+$CD11b$^+$) presented on Fig. 4CD, Fig. 5C and Fig. 6E. (**C**) Gating strategy to detect lung Tfh cells (CD4$^+$B220$^-$ CXCR5$^+$ PD-1$^+$), B cells (CD4$^-$B220$^+$) and Treg cells (CD4$^+$B220$^-$ Foxp3$^+$) presented on Fig. 4ABE, Fig. 5D and Fig. 6F.

Supplementary Figure 2

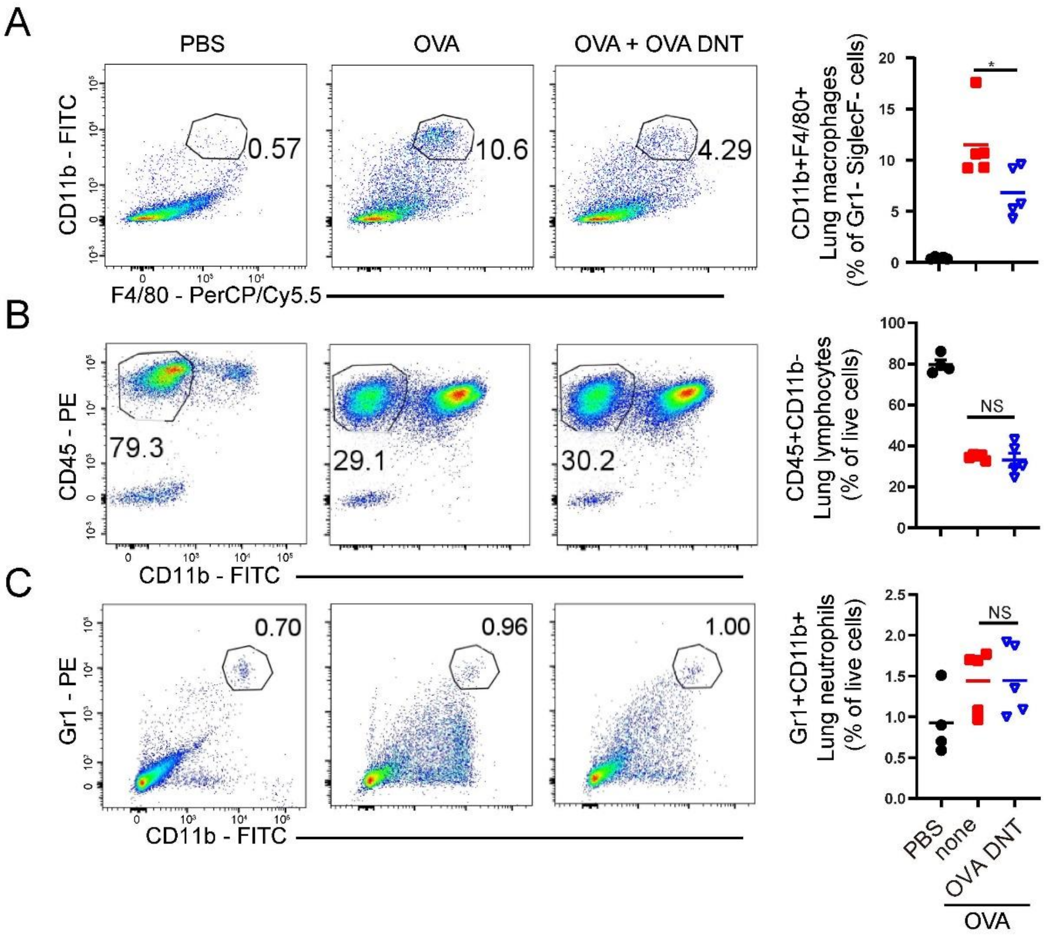

Supplementary Figure 2. The proportion of macrophage, lymphocytes and neutrophils in lungs after DNTs treatment.

Mice received 2 × 10^6 OVA DNTs by intravenous adoptive transfer after the first 1% OVA aerosol challenge on day 28. The mice were challenged daily for the next two days and sacrificed 48 hours after the last aerosol challenge. The percentages of (**A**) macrophages (F4/80^+CD11b^+Gr1^-SiglecF^-), (**B**) lymphocytes (CD45^+CD11b^-) and (**C**) neutrophils (CD11b^+Gr1^+) in the lung were assessed by flow cytometry. Data are shown as the mean ± SEM; n = 4-5 mice per group. One-way ANOVA was used to calculate significance. * $P < 0.05$.

Supplementary Figure 3

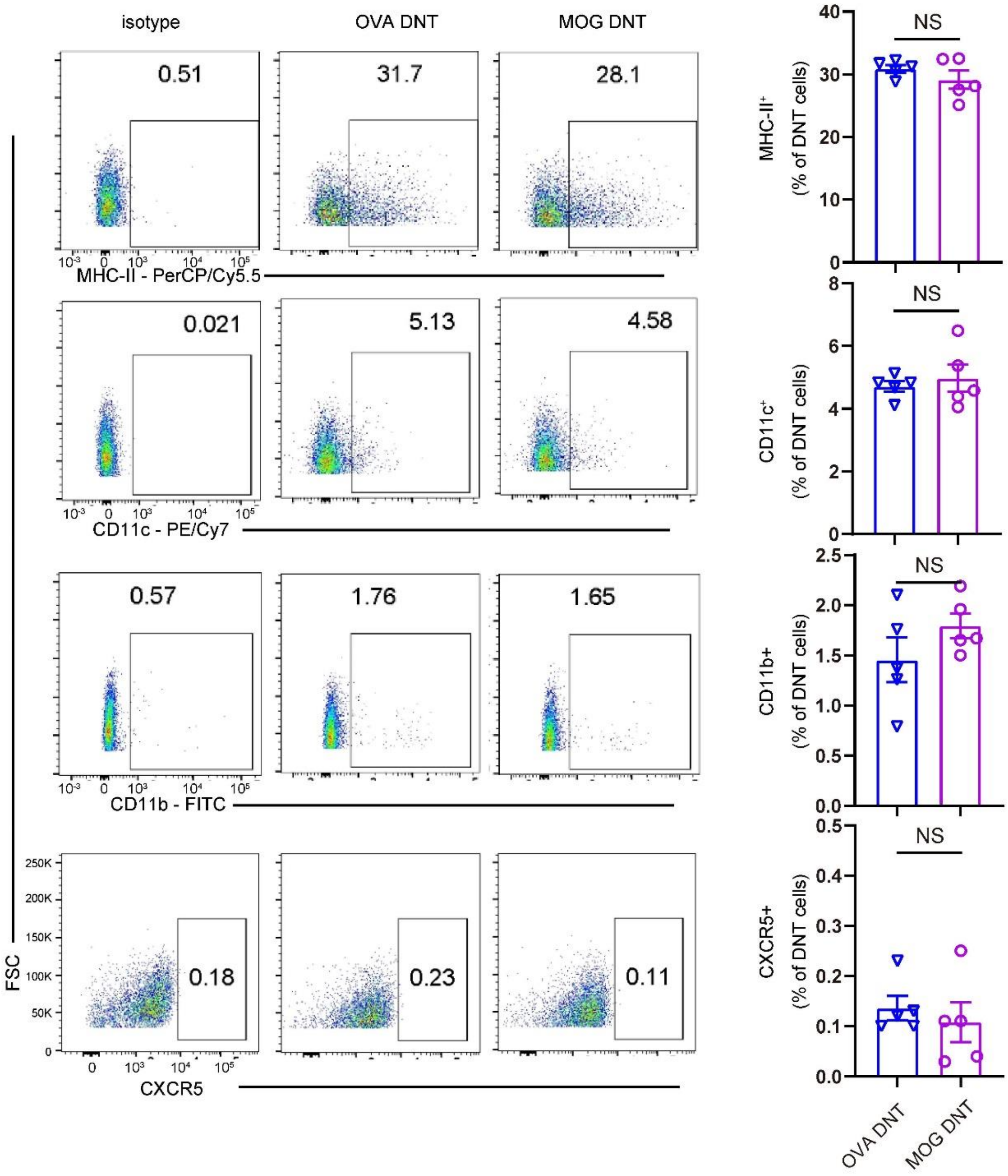

Supplementary Figure 3. MHC-II, CD11c, CD11b and CXCR5 expression of OVA DNT and MOG DNT cells was assessed by flow cytometry. Data are shown as the mean ± SEM; n = 5 mice per group. Student *t* test was used to calculate significance.

Supplementary Figure 4

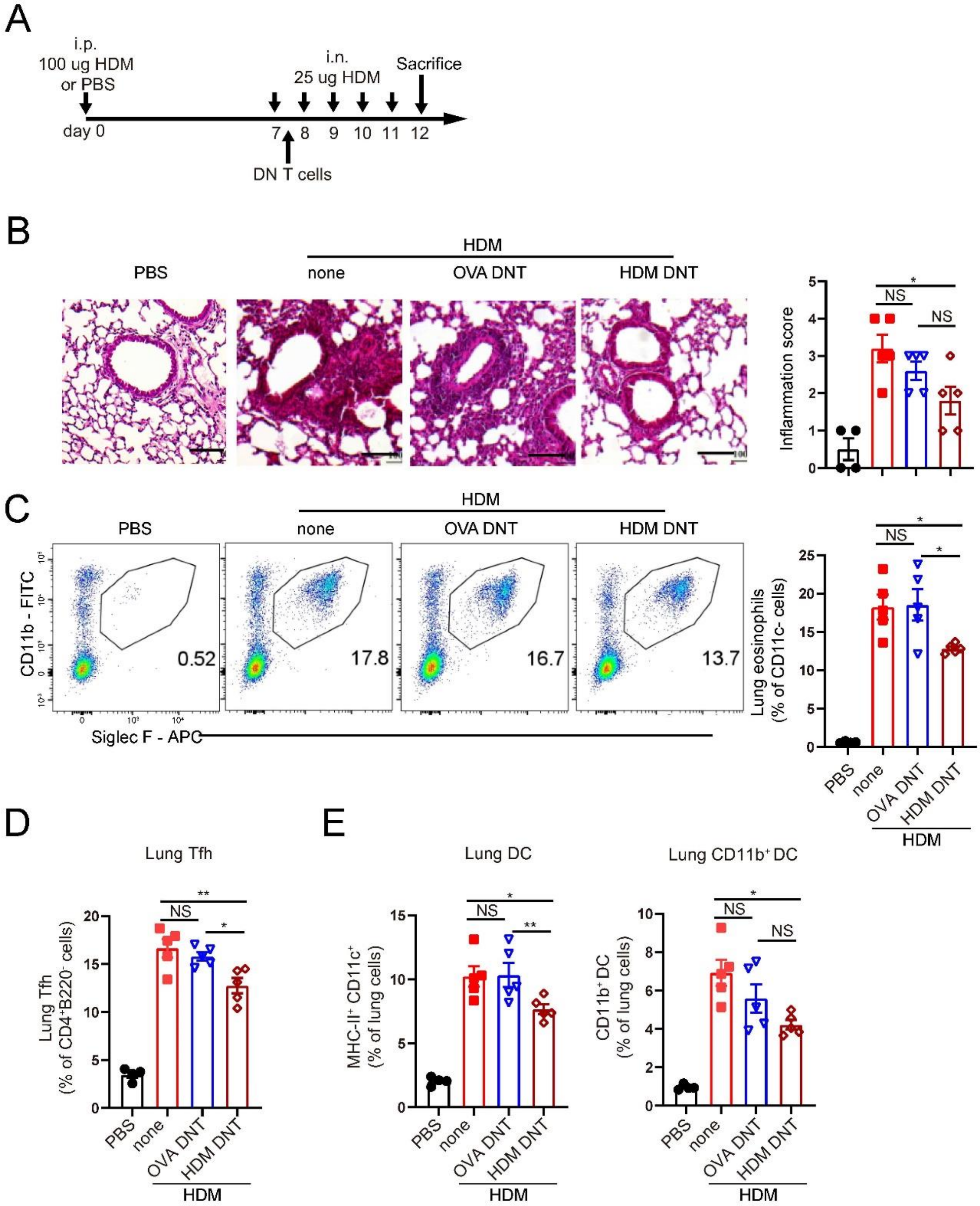

Supplementary Figure 4. The antigen specific therapeutic effect of DNTs in HDM-induced airway inflammation.

(**A**) Schematic representation of the experimental procedure. BALB/c mice were

sensitized by i.p. injection of 100 μg HDM extract. Sensitized mice were administered intranasally with 25 ug of HDM extract daily for 5 days from day 7. *Ex vivo* converted HDM DNT cells or OVA DNT cells (2×10^6) were transferred to mice intravenously after the first i.n. challenge. (**B**) Lung sections were stained with H&E to measure the infiltrated inflammatory cells (Scale bars, 100 μm). The proportions of (**C**) eosinophils (Siglec $F^+CD11b^+CD11c^-$), (**D**)Tfh cells ($CD4^+B220^-CXCR5^+PD\text{-}1^+$) and (**E**) DCs ($CD11c^+MHC\text{-}II^+$) and $CD11b^+$ DCs ($CD11b^+CD11c^+MHC\text{-}II^+$) in the lung were assessed by flow cytometry. Data are shown as the mean ± SEM; n = 4-5 mice per group. One-way ANOVA was used to calculate significance. *$P < 0.05$; **$P < 0.01$.

Supplementary Figure 5

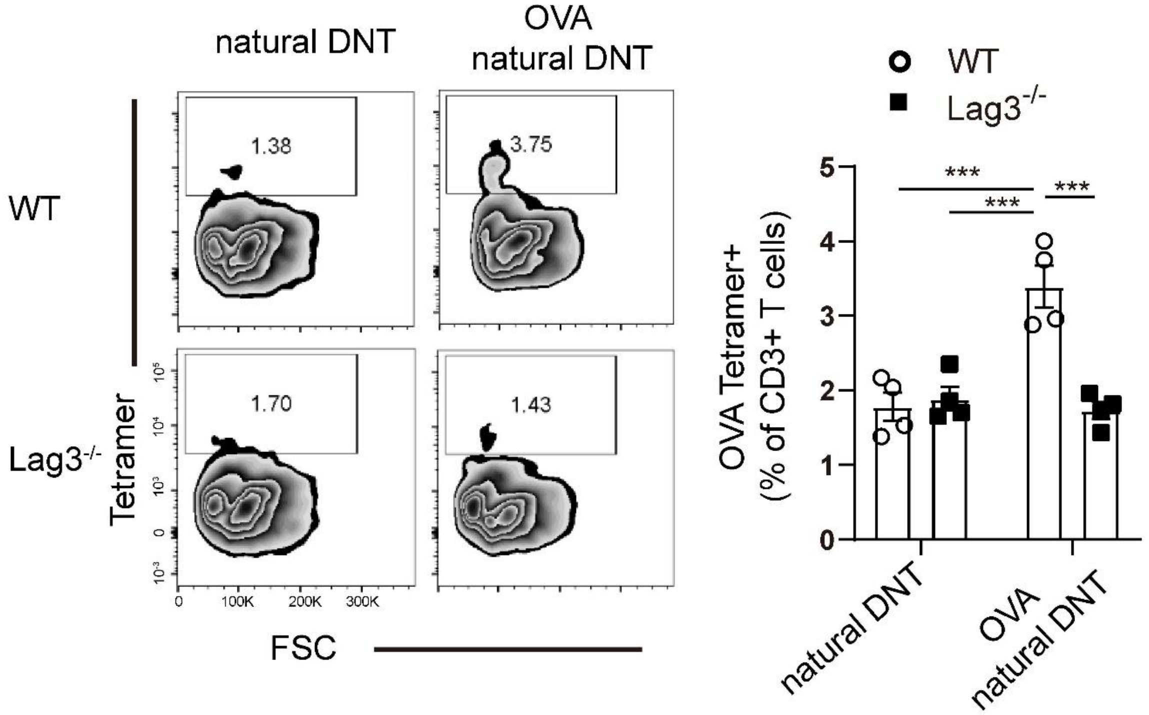

Supplementary Figure 5. OVA-specific natural DNT cells were assessed by OVA-Tetramer-PE staining. Naïve natural DNT cells from WT or Lag3$^{-/-}$ mice were cocultured with C57BL/6J mDCs, 50 ng/ ml rmIL-2 and 1 µg/ml $OVA_{329\text{-}339}$ for 5 days. Activated and freshly isolated naïve WT or Lag3$^{-/-}$ DNT cells were stained with OVA-specific MHC class II tetramer (I-A^{b} $OVA_{323\text{-}339}$ tetramers). OVA-Tetramer^{+} Nature DNT (CD3^{+}OVA-Tetramer^{+}) were assessed by flow cytometry. Data are shown as the mean ± SEM; n = 4 mice per group. One-way ANOVA was used to calculate significance. ***$P < 0.001$.

Supplementary Table 1. Primers used for real-time PCR

Gene name	Sequence (5'→3')
Lag3	F: GAGGCCATCTCGTTCTCGTT
	R: CCGGAAATGGCTGAATCCCA
Prf1	F: CTGCCACTCGGTCAGAATG
	R: CGGAGGGTAGTCACATCCAT
Fasl	F: TGAATTACCCATGTCCCCAG
	R: AAACTGACCCTGGAGGAGCC
β-actin	F: CTGTCCCTGTATGCCTCTG
	R: ATGTCACGCACGATTTCC

F, Forward; R, reverse.
