# Supplementary material for: Double negative T cells mediate Lag3-dependent antigen-specific protection in allergic asthma
Source: Nat Commun. 2019 Sep 18;10:4246. doi: 10.1038/s41467-019-12243-0 (PMC6751182; doi:10.1038/s41467-019-12243-0)
Supplement: Supplementary file 2 — Reporting Summary [file 41467_2019_12243_MOESM2_ESM.pdf]

nature research

Corresponding author(s): Dong Zhang

Last updated by author(s): Aug 16, 2019

Reporting Summary

Nature Research wishes to improve the reproducibility of the work that we publish. This form provides structure for consistency and transparency in reporting. For further information on Nature Research policies, see Authors & Referees and the Editorial Policy Checklist.

Statistics

For all statistical analyses, confirm that the following items are present in the figure legend, table legend, main text, or Methods section.

n/a	Confirmed	
☐	☒	The exact sample size (*n*) for each experimental group/condition, given as a discrete number and unit of measurement
☐	☒	A statement on whether measurements were taken from distinct samples or whether the same sample was measured repeatedly
☐	☒	The statistical test(s) used AND whether they are one- or two-sided<br>*Only common tests should be described solely by name; describe more complex techniques in the Methods section.*
☒	☐	A description of all covariates tested
☐	☒	A description of any assumptions or corrections, such as tests of normality and adjustment for multiple comparisons
☐	☒	A full description of the statistical parameters including central tendency (e.g. means) or other basic estimates (e.g. regression coefficient) AND variation (e.g. standard deviation) or associated estimates of uncertainty (e.g. confidence intervals)
☐	☒	For null hypothesis testing, the test statistic (e.g. *F*, *t*, *r*) with confidence intervals, effect sizes, degrees of freedom and *P* value noted<br>*Give P values as exact values whenever suitable.*
☒	☐	For Bayesian analysis, information on the choice of priors and Markov chain Monte Carlo settings
☒	☐	For hierarchical and complex designs, identification of the appropriate level for tests and full reporting of outcomes
☒	☐	Estimates of effect sizes (e.g. Cohen's *d*, Pearson's *r*), indicating how they were calculated

Our web collection on statistics for biologists contains articles on many of the points above.

Software and code

Policy information about availability of computer code

Data collection	BD FACSDiva was used to collect flow cytometric data.
Data analysis	FlowJo (v10.0.2) was used for flow cytometric analysis. Graphpad Prism was used for statistical analysis.

For manuscripts utilizing custom algorithms or software that are central to the research but not yet described in published literature, software must be made available to editors/reviewers. We strongly encourage code deposition in a community repository (e.g. GitHub). See the Nature Research guidelines for submitting code & software for further information.

Data

Policy information about availability of data

All manuscripts must include a data availability statement. This statement should provide the following information, where applicable:

- Accession codes, unique identifiers, or web links for publicly available datasets
- A list of figures that have associated raw data
- A description of any restrictions on data availability

All data generated and supporting the findings of this study are available within the paper.

Field-specific reporting

Please select the one below that is the best fit for your research. If you are not sure, read the appropriate sections before making your selection.

☒ Life sciences ☐ Behavioural & social sciences ☐ Ecological, evolutionary & environmental sciences

For a reference copy of the document with all sections, see nature.com/documents/nr-reporting-summary-flat.pdf

Life sciences study design

All studies must disclose on these points even when the disclosure is negative.

Sample size	A reasonable sample size was chosen based on our previous studies and similar literatures to ensure adequate reproducibility of results.
Data exclusions	None.
Replication	Experimental findings were reproducible across multiple experiments.
Randomization	Animals were randomly allocated into experimental groups.
Blinding	We were blinded to group allocation during data collection and/or analysis.

Reporting for specific materials, systems and methods

We require information from authors about some types of materials, experimental systems and methods used in many studies. Here, indicate whether each material, system or method listed is relevant to your study. If you are not sure if a list item applies to your research, read the appropriate section before selecting a response.

Materials & experimental systems

n/a	Involved in the study	
☐	☒	Antibodies
☒	☐	Eukaryotic cell lines
☒	☐	Palaeontology
☐	☒	Animals and other organisms
☒	☐	Human research participants
☒	☐	Clinical data

Methods

n/a	Involved in the study	
☒	☐	ChIP-seq
☐	☒	Flow cytometry
☒	☐	MRI-based neuroimaging

Antibodies

Antibodies used	The following antibodies were used for flow cytometry: Anti-CD11b (ICRF44), anti-CD11c (N418), anti-I-A/I-E (M5/114.15.2), anti-CD8a (53-6.7), anti-CD103 (M290), anti-CD40 (3/23), anti-CD80 (16-10A1), anti-Siglec F (E50-2440), anti-Ly-6G (RB6-8C5), Anti-CD4 (GK1.5), anti-B220 (RA3-6B2), anti-CXCR5 (SPRCL5), anti-Foxp3 (MF-14), anti-GzmB (GB11), anti-PD-1 (29F.1A12), anti-IL4 (11B11), anti-IL-13 (eBio13A), anti-IL-17A (TC11-18H10), anti-IL-21 (FFA21) and anti-IFN-γ (XMG1.2).<br>The following antibodies were used for confocal microscopy: anti-Lymphocyte Activation Gene 3 (EPR20294-77) and anti-I-Ab (AF6-120.1).
Validation	The antibody validation is provided on supplier website.

Animals and other organisms

Policy information about studies involving animals; ARRIVE guidelines recommended for reporting animal research

Laboratory animals	The following strains were used: C57BL/6J, C57BL/6J-GFP, BALB/c and Lag3-/-. Mice were between the ages of 8-12 weeks old.
Wild animals	N/A
Field-collected samples	N/A
Ethics oversight	All procedures were performed in accordance with the guidelines set by the Institutional Animal Care and Ethics Committee at Beijing Friendship Hospital.

Note that full information on the approval of the study protocol must also be provided in the manuscript.

Flow Cytometry

Plots

Confirm that:

☒ The axis labels state the marker and fluorochrome used (e.g. CD4-FITC).

☒ The axis scales are clearly visible. Include numbers along axes only for bottom left plot of group (a 'group' is an analysis of identical markers).

☒ All plots are contour plots with outliers or pseudocolor plots.

☒ A numerical value for number of cells or percentage (with statistics) is provided.

Methodology

Sample preparation	Mice were sacrificed by cervical dislocation. PBS was injected into the right ventricle to flush circulating blood cells. Lungs were chopped to small pieces and digested with tissue digestion solution (collagenase IV 0.5 mg/ml plus DNase I 8 ug/ml in HBSS containing 5% FBS). Tissues were gently homogenized with a 1-mL syringe plunger, and cells were dissociated through a 70-um cell strainer (BD Biosciences). RBC lysis buffer (Qiagen) was used to lyse red blood cells.
Instrument	FACSAriaII (BD Biosciences)
Software	FlowJo software (Tree Star)
Cell population abundance	Cell populations were sorted to >95% purity post sort in pilot experiments, as determined by flow cytometry.
Gating strategy	Eosinophils were gated as Siglec F+CD11b+CD11c-; Tfh cells were gated as CD4+B220-CXCR5+PD-1+; Treg cells were gated as CD4+B220-Foxp3+; CD11b+ DCs were gated as CD11c+MHC-II+CD11b+; CD103+ DCs were gated as CD11c+MHC-II+CD103 +CD11b-; B cells were gated as B220+CD4-; macrophages were gated as F4/80+ CD11b+ Gr1- SiglecF-; lymphocytes were gated as CD45+ CD11b-; neutrophils were gated as CD11b+ Gr1+.

☒ Tick this box to confirm that a figure exemplifying the gating strategy is provided in the Supplementary Information.
